# Commentary: The efficacy of ginseng-containing traditional Chinese medicine in patients with acute decompensated heart failure: a systematic review and meta-analysis

**DOI:** 10.3389/fphar.2023.1180944

**Published:** 2023-08-01

**Authors:** Niansong Kang, Sihui Zheng

**Affiliations:** ^1^ Department of Traditional Chinese Medicine, Yuyao Hospital of Traditional Chinese Medicine, Yuyao, Zhejiang, China; ^2^ Department of Gastroenterology, The First Clinical Medical College of Zhejiang Chinese Medicine University, Hangzhou, Zhejiang, China

**Keywords:** ginseng, acute decompensated heart failure, meta-analysis, commentary, review

The most prominent limitation of this meta-analysis is the inaccuracy of the literature search. In this article, the author emphasized that seven commonly used Chinese and English databases were searched, and the search time point was before July 2022. Finally, 12 articles related to “Shenfu Injection” were included. However, it must be recognized that randomized controlled studies on Shenfu injection and acute heart failure are very abundant in China. Therefore, through the keywords provided by the authors, we reconducted an extensive search for studies related to “Shenfu injection.” Not surprisingly, four eligible studies were omitted by the authors (Tao and Fu, 2013; Jiang et al., 2014; Chen, 2015; Tang and Chu, 2017). Although the conclusions of these five articles are consistent with the authors, a more adequate sample size allows the authors to conduct a deeper analysis of the sources of high heterogeneity in the outcomes. In addition, the authors need to further expand the English database to find English papers as much as possible, and only 3 English databases may miss high-quality target literatures. For example, the authors could add the following English databases: Scopus, Google Scholar, Web of Science, Cochrane Library, and LitCovid.

The high degree of heterogeneity in the primary outcome is another drawback of this meta-analysis. It must be acknowledged that in order to explain the source of heterogeneity, the authors performed valid subgroup analyses according to three possible factors. We would like to provide constructive comments on this massive project to further refine and consolidate the findings of the study. The authors performed subgroup analyses according to treatment period (within or beyond 2 weeks). Whether it is possible to further explore the possible time-effect relationship for some outcomes? For example, BNP or NT-proBNP levels may show a more pronounced trend over time. Secondly, considering the difference of drug dose (such as Shenfu Injection), can we explore the optimal dose for patients with ADHF by adding the missing studies? Third, the results would be further improved if the authors could report left ventricular fractional shortening and TCM syndrome scores. Finally, we recommend using the inverse variance heterogeneity model to verify the stability of highly heterogeneous outcomes in order to dispel the widespread distrust of high heterogeneity (Doi et al., 2015).
